# Meis2 Is Required for Inner Ear Formation and Proper Morphogenesis of the Cochlea

**DOI:** 10.3389/fcell.2021.679325

**Published:** 2021-05-28

**Authors:** María Beatriz Durán Alonso, Victor Vendrell, Iris López-Hernández, María Teresa Alonso, Donna M. Martin, Fernando Giráldez, Laura Carramolino, Giovanna Giovinazzo, Enrique Vázquez, Miguel Torres, Thomas Schimmang

**Affiliations:** ^1^Instituto de Biología y Genética Molecular, Universidad de Valladolid y Consejo Superior de Investigaciones Científicas, Valladolid, Spain; ^2^Departments of Pediatrics and Human Genetics, University of Michigan, Ann Arbor, MI, United States; ^3^CEXS, Universitat Pompeu Fabra, Parc de Recerca Biomédica de Barcelona, Barcelona, Spain; ^4^Cardiovascular Development Program, Centro Nacional de Investigaciones Cardiovasculares, CNIC, Madrid, Spain

**Keywords:** inner ear, cochlea, Meis, organ of corti, mouse

## Abstract

*Meis* genes have been shown to control essential processes during development of the central and peripheral nervous system. Here we have explored the roles of the *Meis2* gene during vertebrate inner ear induction and the formation of the cochlea. *Meis2* is expressed in several tissues required for inner ear induction and in non-sensory tissue of the cochlear duct. Global inactivation of *Meis2* in the mouse leads to a severely reduced size of the otic vesicle. Tissue-specific knock outs of *Meis2* reveal that its expression in the hindbrain is essential for otic vesicle formation. Inactivation of *Meis2* in the inner ear itself leads to an aberrant coiling of the cochlear duct. By analyzing transcriptomes obtained from *Meis2* mutants and ChIPseq analysis of an otic cell line, we define candidate target genes for *Meis2* which may be directly or indirectly involved in cochlear morphogenesis. Taken together, these data show that *Meis2* is essential for inner ear formation and provide an entry point to unveil the network underlying proper coiling of the cochlear duct.

## Introduction

Development of the inner ear begins as a thickening of the ectoderm adjacent to the posterior hindbrain termed the otic placode, which can be observed at embryonic day 8 (E 8) in the mouse. In vertebrates, induction of the otic placode requires the interaction with neighboring tissues such as the neural tissue of the hindbrain, the mesoderm and/or the endoderm. Members of the fibroblast growth factor (*Fgf*) gene family such as *Fgf3* have been shown to play a central role during otic placode induction (Schimmang, 2007; Whitfield, 2015). After E8 in the mouse, the otic placode invaginates and forms the otic vesicle, which undergoes a series of morphogenetic steps to form the complex shape of the mature inner ear. Cochlear morphogenesis is initiated at the ventral portion of the otic vesicle that elongates and coils in an anterior–medial direction until it reaches its full one and three−quarters turns. This process is paralleled by cellular differentiation that leads to the formation of sensory cells such as hair cells and auditory neurons, and non-sensory components within the cochlear duct (Basch et al., 2016).

*Meis* genes are vertebrate orthologs of the Drosophila homolog *homothorax* (*hth*) gene which encode for transcription factors belonging to the superclass of TALE (three amino acid loop extension) proteins. *Meis* genes play key roles during development of the central and peripheral nervous systems, and interact with signaling pathways such as those controlled by Wnt, Fgf and retinoic acid (Schulte and Frank, 2014). The TALE superclass of proteins contains an atypical homeodomain and comprises five separate classes: Meinox, including Prep (*Prep1–2* genes) and Meis (*Meis1–3* genes), Pbc (*Pbx1–4* genes) and the three more distantly related TG-interacting factors, Iroquois, and Mohawk (Schulte and Geerts, 2019). A prominent characteristic of Meinox proteins is their capacity to heterodimerize with the structurally related Pbx proteins. A further group of Meis protein-binding partners participating in these cooperative interactions is the Hox proteins. In this case, Meis proteins often do not interact directly with DNA (Penkov et al., 2013).

In the present work, we have analyzed the role of *Meis2* during inner ear induction and cochlear development. We show that during inner ear induction *Meis2* is prominently expressed in the hindbrain neighboring the otic placode and to a lesser extent in the periotic mesoderm and endoderm. Specific inactivation in the rhombomeres flanking the otic placode, led to reduced otic vesicles, uncovering a dominant role of hindbrain *Meis2* in otic vesicle formation. Inactivation of *Meis2* within the otic placode caused morphological defects including improper coiling of the cochlea. Microarray analysis revealed a set of genes that were downregulated in mutant cochleas, representing potential *Meis2* target genes required for proper morphogenesis of the cochlear duct. Finally, ChIPseq analysis in an otic cell line with the potential to give rise to sensory and non-sensory cochlear tissue (VOT-E36) allowed the detection of genes whose regulatory regions bind Meis2 directly or indirectly. Our results thus show that *Meis2* is essential for otic vesicle formation and cochlear duct morphogenesis.

## Results

### *Meis2* Expression During Inner Ear Development

**FIGURE 1:**
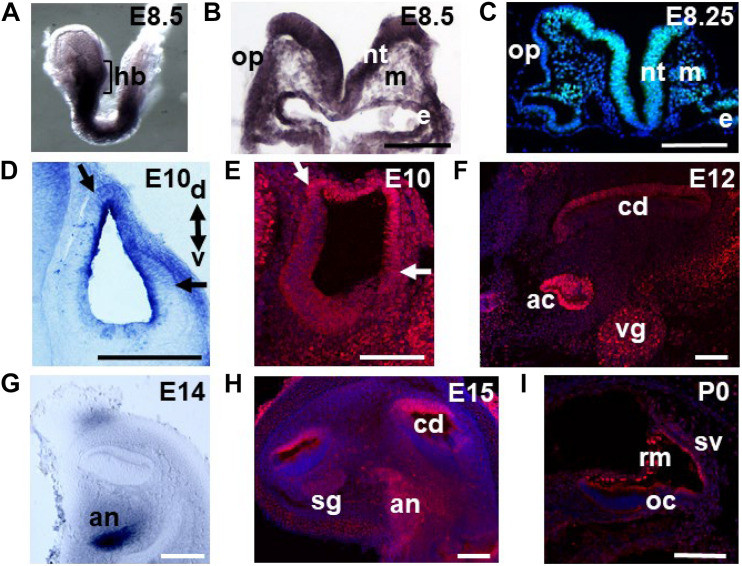
Meis2 expression throughout inner ear development. Meis2 expression was detected via mRNA *in situ* hybridization **(A,B,D,G)** or immunohistochemistry **(C,E,F,H,I)** at the indicated stages. **(A)** At embryonic day (E) 8.5 high levels of Meis2 are detected at the level of the hindbrain (hb) and on corresponding sections at the level of the otic placode (op) **(B)**. High levels of Meis2 protein are detected in the neural tube (nt), the endoderm (e), and weaker expression in the mesoderm (m) and the placode itself **(C)**. **(D)** In the otic vesicle low *Meis2* mRNA levels are observed in its dorsal-lateral portion (borders indicated by arrows). The orientations of the sections through the otic vesicles along the dorsal (d)–ventral (v) axis are indicated. **(E)** Immunohistochemical detection of Meis2 protein in the dorsal-lateral quadrant of the otic vesicle. **(F)** Presence of Meis2 protein in the roof of the cochlear duct (cd), the vestibular ganglion (vg) and the ampullary cristae (ac). **(G)** High levels of *Meis2* mRNA are detected in the auditory nerve (an). **(H)** Next to the auditory nerve, Meis2 protein is also observed in the roof of the cochlear duct and to a lesser extent in the spiral ganglion (sg). **(I)** At P0 Meis2 immunoreactivity is detected in Reissner’s membrane (rm) and the stria vascularis (sv). oc, organ of Corti. Scale bars in **(B,C,E–H)**: 100 μm; in **(D)**: 200 μm and in **(I)**: 75 μm.

In order to define *Meis2* expression throughout inner ear development, we performed whole mount RNA *in situ* hybridization and immunohistochemistry studies. During inner ear induction around E8–8.5, high levels of mRNA were detected in the developing hindbrain (Figure 1A). Sections of the posterior hindbrain confirmed this expression and revealed *Meis2* transcripts in the otic placode and the neighboring mesoderm and endoderm (Figure 1B). Immunohistochemistry confirmed high levels of Meis2 protein in the hindbrain and moderate to low amounts in the endoderm, mesoderm and otic placode (Figure 1C). Upon formation of the otic vesicle Meis2 immunoreactivity was observed in its dorsolateral domain, accompanied by low but detectable *Meis2* mRNA (Figures 1D,E).

During inner ear morphogenesis around E12, Meis2 protein was detected in the roof of the cochlear duct, which will give rise to non-sensory tissue (Figure 1F). Expression in this domain was maintained at E15 and was also detected in the auditory nerve at the protein and shortly before at the mRNA level (Figures 1G,H). Low levels of anti-Meis2 immunoreactivity were also observed in the spiral ganglion (Figure 1H). At postnatal day 0 (P0), Meis2 protein was observed in Reissner’s membrane and the stria vascularis but not in the sensory tissue corresponding to the organ of Corti (Figure 1I). Adjacent to the cochlea, Meis2 protein was also detected in different parts of the vestibular system such as the ampullary cristae, the utriculus, the semicircular canals and the vestibular ganglion (Figure 1F and data not shown).

### Meis2 Is Required for Otic Vesicle Formation

**FIGURE 2:**
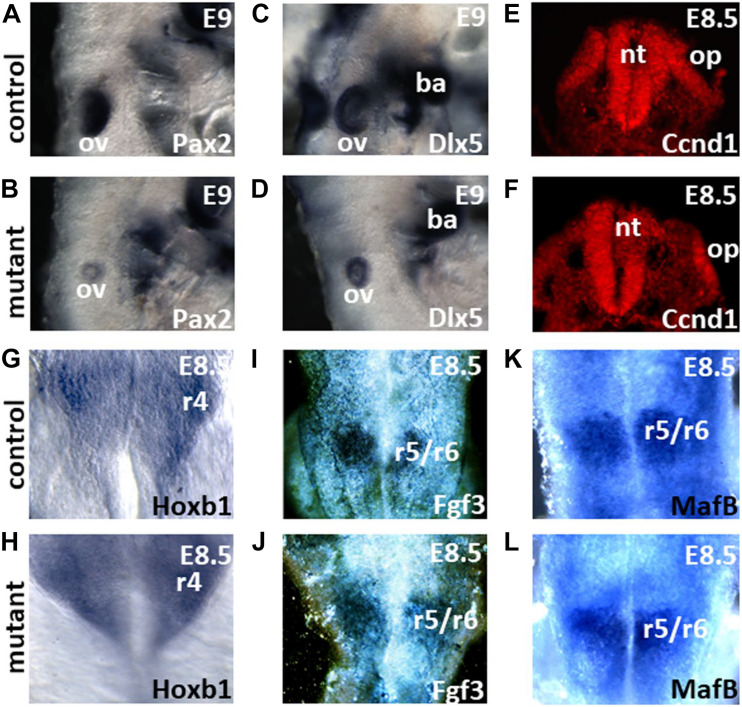
Global inactivation of *Meis2* affects inner ear formation. **(A–D)** *Meis2*^flox/flox^; *Sox2*^Cre/+^ mutants show severely reduced otic vesicles (ov) at embryonic day 9 (E9) in comparison to controls as revealed by *Pax2* and *Dlx5* riboprobes. **(E,F)** Sections reveal widespread cyclinD1 immunoreactivity in the neural tube (nt) and the otic placode (op) of controls and mutants. Note the reduced size of the otic placode in the mutant. **(G–L)** Flatmounts of the hindbrain reveal an unaltered expression of *Hoxb1, Fgf3* and *MafB* riboprobes in the indicated rhombomeres (r) in *Meis2*^flox/flox^; *Sox2*^Cre/+^ mutants in comparison to control embryos. ba, branchial arch.

To analyze the requirement of *Meis2* for inner ear formation, we inactivated its expression throughout the epiblast by crossing a mouse strain carrying a floxed *Meis2* allele with a *Sox2-*Cre deleter strain (Hayashi et al., 2002). *Meis2*^flox/flox^; *Sox2*^Cre/+^ embryos showed a severely reduced otic vesicle at E9 as revealed by *in situ* hybridization with the otic markers *Pax2* and *Dlx5* (Figures 2A–D), confirming similar observations made in *Meis2* null mutants (Machon et al., 2015). Therefore, *Meis2* is required for otic vesicle formation.

**FIGURE 3:**
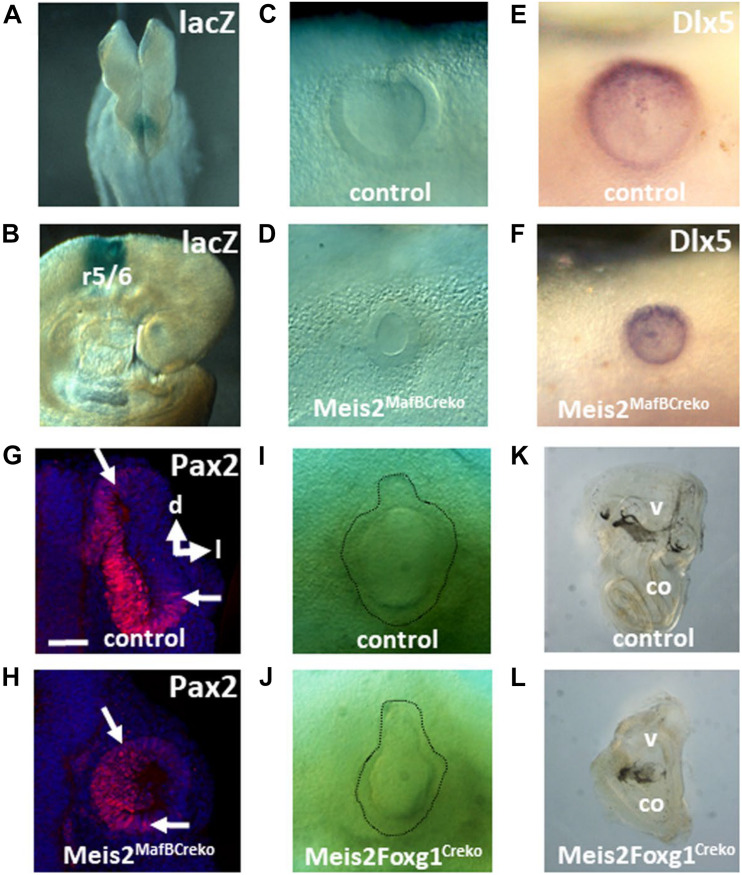
Effects of tissue-specific inactivation of Meis2 on inner ear formation. **(A,B)** lacZ *ROSA*26 reporter staining caused by the *MafB*Cre driver in the posterior hindbrain of E8.25 embryos and in rhombomeres (r) flanking the otic vesicle at E9 **(B)**. **(C–H)** Meis2^flox/flox^; *MafB*^Cre/+^ mutants show reduced sized vesicles **(C,D)** which maintain expression of *Dlx5* (at E9) and Pax2 (at E9,5), as revealed by whole mount RNA *in situ* hybridization **(E,F)** and immunohistochemistry **(G,H)**. The orientations of the sections through the otic vesicles along the dorsal (d)–lateral (l) axis are indicated. **(I–L)** Bright field view of otic vesicles at E11.5 and cleared inner ears at P1 from *Meis2*^flox/flox^; *Foxg1*^Cre/+^ mutants. Note the flask-shaped morphology of the otic vesicle and the lack of a discernible cochlea (co) in the mutants. v, vestibule.

Next, we were interested to determine the tissue-specific requirements for *Meis2* during otic induction. Since *Meis2* was strongly expressed in the hindbrain during otic development, we sought to find a Cre line with a specific activity in the hindbrain. The *MafB* gene is expressed in the posterior hindbrain next to the developing otic placode and vesicle (Cordes and Barsh, 1994). Recently, a *MafB-*Cre line has been employed for lineage tracing of macrophages which are also characterized by endogenous *MafB* expression (Wu et al., 2016). To assay if Cre activity also occurred in the developing hindbrain, we crossed *MafB*-Cre transgenic mice with *ROSA26* reporter mice (Soriano, 1999). Cre activity was detected by X-gal staining at E8.5 in the posterior hindbrain and in rhombomeres flanking the otic vesicle at E9 (Figures 3A,B and Supplementary Figure 1). This confirmed its utility for the inactivation of floxed genes during inner ear formation. In order to study the requirement for hindbrain *Meis2* expression during otic vesicle formation, we analyzed the effects of *Meis2* conditional inactivation induced by *MafB*-Cre at the otic vesicle stage. *Meis2*^flox/flox^; *MafB*^Cre/+^ mutants showed a severely reduced vesicle at E9 comparable to the size observed upon global *Meis2* inactivation with a penetrance of 50% (*n* = 2/4; Figures 3C,D). Therefore Meis2 expression in the hindbrain is required for otic vesicle formation. Staining with the otic markers *Dlx5* and *Pax2* revealed that their expression was maintained in their corresponding domains in *Meis2*^flox/flox^; *MafB*^Cre/+^ mutants (Figures 3E–H). To analyze the influence of Meis2 expression in neighboring tissue during otic induction, we used a *Foxg1*-Cre line which next to the otic placode also drives expression in peri-otic mesoderm and endoderm (Zelarayan et al., 2007; Supplementary Figure 1). Conditional inactivation of *Meis2* using the *Foxg1*-Cre line did not affect inner ear formation until E11.5 when a reduced sized otic vesicle with a flask-like shape was observed in *Meis2*^flox/flox^; *Foxg1*^Cre/+^ mutants (Figures 3I,J). *Meis2*^flox/flox^; *Foxg1*^Cre/+^ inner ears isolated at P0 and cleared with methylsalicylate revealed no discernible structures such as the cochlea or the semicircular canals (Figures 3K,L).

With the aim to define the molecular changes underlying defective inner ear induction, we analyzed *Meis2*^flox/flox^; *Sox2*^Cre/+^ mutants which show severely reduced sized otic vesicles with a penetrance of 100% for alterations in gene expression or signaling pathways known to be controlled by *Meis* genes and related to inner ear formation. Formation of the hindbrain requires *Meis* gene expression in lower vertebrates and has been shown to activate *Fgf3* expression which is redundantly required together with other *Fgf* family members to induce the otic placode in vertebrates (Maroon et al., 2002; Alvarez et al., 2003; Wright and Mansour, 2003; Ladher et al., 2005; Gutkovich et al., 2010). Moreover, *Fgf3* has been shown to participate in the induction of *MafB* (Zelarayan et al., 2007), and *MafB* inactivation also leads to reduced sized otic vesicles (Cordes and Barsh, 1994; Wiellette and Sive, 2003; Hernandez et al., 2004). Lastly, Meis proteins together with Fgf signaling have also been shown to induce in the rhombencephalon expression of *Hox* genes that belong to the paralogous group 1 (PG1) and which are likewise required for in inner ear induction (Rossel and Capecchi, 1999; Pasqualetti et al., 2001; Schulte and Frank, 2014). RNA *in situ* hybridization with probes corresponding to *Fgf3, MafB* and the PG1 gene *HoxB1* showed no changes in their expression pattern within rhombomeres flanking the developing otic placode in *Meis2*^flox/flox^; *Sox2*^Cre/+^ mutants (Figures 2G–L).

Alternatively to hindbrain patterning defects, reduced proliferation may cause the smaller sized vesicles. Meis has been shown to regulate cyclinD1 and thereby to control eye size in vertebrates (Bessa et al., 2008; Marcos et al., 2015). CyclinD1 was prominently expressed in the hindbrain and the neighboring otic placode of both controls and *Meis2*^flox/flox^; *Sox2*^Cre/+^ mutants (Figures 2E,F).

### Conditional Inactivation of *Meis2* During Inner Ear Development Affects Cochlear Coiling

**FIGURE 4:**
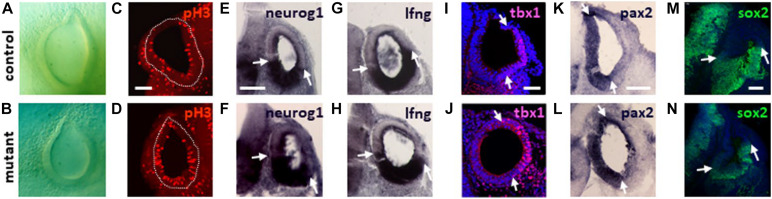
Expression of otic vesicle markers in *Meis2*^flox/flox^; *Pax2*^Cre/+^ mutants. Expression of the indicated markers was detected via mRNA *in situ* hybridization **(E–H,K,L)** or immunohistochemistry **(C,D,I,J,M,N)**. **(A,B)** Bright field image of controls and mutants showing a reduced sized otic vesicle at E10,5. **(C,D)** Sections at E10 reveal widespread pH3 immunoreactivity in the otic vesicles (circumference indicated by stippled lines) of controls and mutants. **(E–L)** No changes in the spatial domains of the otic markers (borders indicated by arrows) is observed in the mutants at E9 **(E–H)** and E10 **(I–L)**. **(M,N)** At E11 the ventral domain of Sox2 expression is smaller in mutants correlating with the reduced sized dimensions of the otic vesicle at this stage. Scale bars in **(C,E,I)**: 50 μm; in **(K)**: 100 μm and in **(M)**: 75 μm.

To analyze the function of *Meis2* expression in the inner ear, we generated conditional mutant mice using a Cre line driven by *Pax2* regulatory sequences which has been used to inactivate floxed alleles throughout inner ear development (Ohyama and Groves, 2004; Supplementary Figure 1). No morphological defects in inner ear formation were found until E10.5 in *Meis2*^flox/flox^; *Pax2*^Cre/+^ mutants, when a reduced sized otic vesicle was observed in comparison to controls (Figures 4A,B).

The smaller size of the otic vesicle observed in *Meis2*^flox/flox^; *Pax2*^Cre/+^ mutants may be caused by a lack of proliferation. To examine cell proliferation, we used staining with an antibody against pH3 that labels cells in late G2 and M phase of the cell cycle. Immunoreactivity for pH3 was observed throughout the otic epithelium of both wild-type and *Meis2*^flox/flox^; *Pax2*^Cre/+^ mutants (Figures 4C,D). To examine potential changes in molecular markers in mutant otic vesicles, we first examined the neurosensory region. This develops in an anterior-medial domain of the otic vesicle and is characterized by the expression of *Neurogenin 1* (*Ngn1*) and *Lunatic fringe* (*Lfng*). Complementary to *Ngn1* expression, genes like *Tbx1* (*T-box transcription factor 1*) stabilize the neurogenic region (for a review, see Bok et al., 2007). No difference in the expression patterns of these markers was observed in *Meis2*^flox/flox^; *Pax2*^Cre/+^ mutants when compared to control otic vesicles (Figures 4E–J).

**FIGURE 5:**
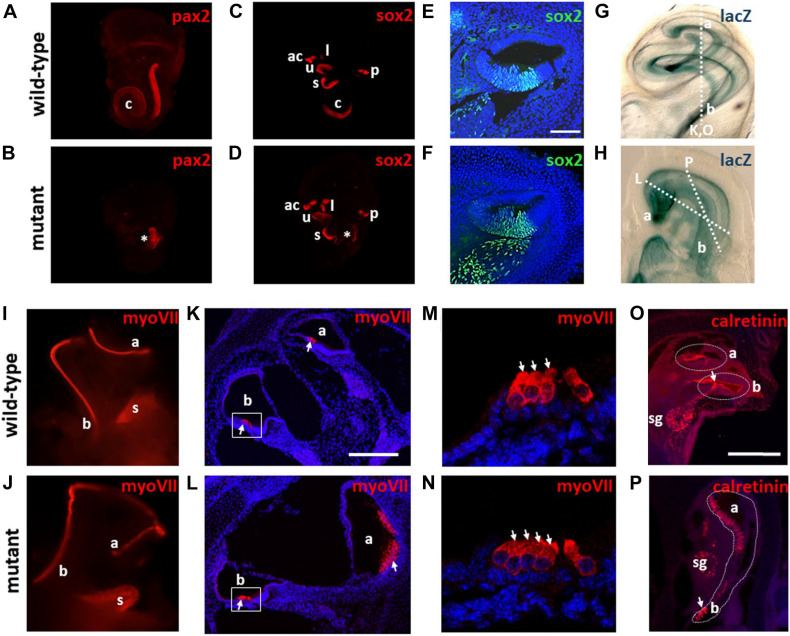
Meis2 is required for cochlear outgrowth and coiling. Expression was monitored by staining of whole mounts or on sections with the corresponding antibodies indicated. **(A–D)** Staining with Pax2 and Sox2 antibodies reveals a truncation of the cochlear duct (c), indicated by an asterisk in the mutants. Note that the prosensory regions within the vestibular system maintain Sox2 expression in the mutants. **(E,F)** Sections through the cochlear duct reveal that formation of the prosensory region is unaffected in the mutants. **(G,H)** lacZ*ROSA26* reporter staining caused by the *Pax2*-Cre driver reveals the abnormal coiling of the cochlear duct in mutants. The basal (b) and apical (a) portions of the cochlea are indicated. **(I–L)** Staining with myosin VII antibodies labeling hair cells (indicated by arrows) confirms the abnormal morphology of the cochlear duct in mutants. Note the increased sized patches of hair cells in the apex of mutants. **(M,N)** High magnification view of the basal region (boxed in **K,L**) reveals the presence of an extra row of outer hair cells (indicated by arrows) in the mutants. **(O,P)** Sections stained with calretinin antibodies labeling inner hair cells (arrows) and the spiral ganglion (sg). Note the presence of a normal morphology in the wild-type with the typical appearance of basal and apical turns whereas the mutant shows an abnormal extension along the basal-apical axis. The plane of the sections shown in **(K,L,O,P)** are indicated in **(G,H)**. ac, anterior cristae; l, lateral cristae; p, posterior cristae; s, sacculus; u, utriculus; Scale bars: 50 μm in **(E)**; 250 μm in **(K,O)**.

During normal development, *Pax2* expression is localized in the medial wall of the otic vesicle (Figure 4K) whereas the ventral portion is characterized by a broad domain of Sox2 at E11.5 from where the cochlear anlage derives (Basch et al., 2016; Figure 4M). Later on, *Pax2* and *Sox2* label the non-sensory and pro-sensory regions within the developing cochlear duct, respectively (Burton et al., 2004; Mak et al., 2009; Figures 5A,C). *Pax2* showed a normal pattern of expression at the otic vesicle stage in *Meis2*^flox/flox^; *Pax2*^Cre/+^ mutants (Figure 4L) but its domain was severely reduced at E14, pointing to a potential truncation of the cochlear duct (Figure 5B). *Sox2* staining at E11.5 revealed a reduced expression domain that correlated with the smaller size of the otic vesicle in *Meis2*^flox/flox^; *Pax2*^Cre/+^ mutants (Figure 4N). At E14 we observed a small patch of Sox2 expression at the basal portion of the cochlea, confirming the shortening of the cochlear duct (Figure 5D). However, sections through the cochlear duct revealed that the formation of the prosensory region in *Meis2*^flox/flox^; *Pax2*^Cre/+^ mutants was unaffected (Figures 5E,F). Sox2 staining was also unaffected in the prosensory region of all vestibular sensory epithelia, including the utricular and saccular maculae and posterior, lateral, and anterior cristae (Figures 5C,D).

To further explore the cochlear abnormality in *Meis2*^flox/flox^; *Pax2*^Cre/+^ mice, we performed whole-mount β-galactosidase staining of the inner ears from mutants which also carried a *ROSA26*lacZ reporter (Soriano, 1999). *Pax2*-Cre is active in the early otic placode and vesicle, and the *ROSA26*lacZ reporter allows labeling of all inner ear components throughout development (Ohyama and Groves, 2004). Beta-galactosidase staining revealed that in control animals, the cochlea had undertaken its one and three quarter turns (Figure 5G). In contrast, in *Meis2*^flox/flox^; *Pax2*^Cre/+^ mutants, instead of turning ventrally along the anterior-medial axis, the cochlear duct extended toward the apex but then took a U-turn toward the base. This lead to its termination being positioned half-way from its point of initiation (Figure 5H). This irregular turning was also confirmed by whole mount staining with myosin VII antibodies which label sensory hair cells within the cochlear duct (Figures 5I,J). Sections through control ears confirmed the typical structure of the cochlear duct including one row of inner hair cells and three rows of outer hair cells in controls (Figures 5K,M). In contrast, sections from *Meis2*^flox/flox^; *Pax2*^Cre/+^ mutant cochleas revealed an extra row of outer hair cells in the basal turn and an enlarged cochlear duct with clusters of hair cells at the apex (Figures 5L,N). Staining with calretinin antibodies that label spiral ganglion neurons and inner hair cells confirmed a normally structured cochlea with the typical appearance of basal and apical turns in control animals, whereas sections of *Meis2*^flox/flox^; *Pax2*^Cre/+^ mutants revealed an abnormal extension of the cochlear duct along the basal to apical axis (Figures 5O,P). In summary, these data confirm that loss of *Meis2* during inner ear development leads to a defective cochlear coiling and extra rows of hair cells.

### Direct and Indirect Targets of Meis2 in the Cochlea

**FIGURE 6:**
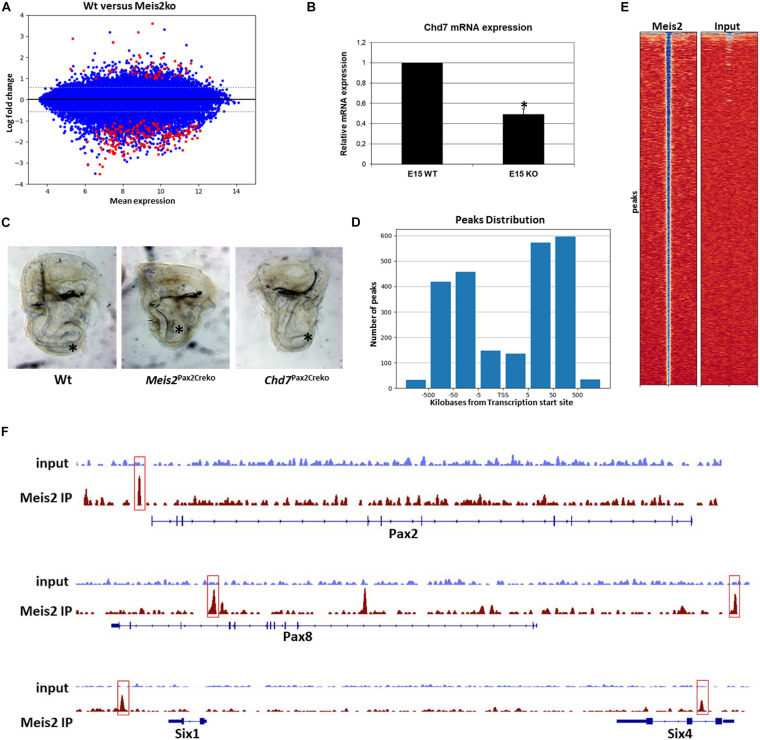
Targets of Meis2. **(A)** MA plot showing differential gene expression between *Meis2*^flox/flox^; *Pax2*^Cre/+^ mutant vs. wild-type cochleas at E15. Red dots indicate significantly regulated genes. **(B)** Significantly reduced expression of *Chd7* is observed in the cochlea of *Meis2*^flox/flox^; *Pax2*^Cre/+^ mutants (KO) by qPCR at E15. Data were normalized to expression levels in wild-type (WT) cochleas. Student’s *t*-test: *p < 0.0001 **(C)** *Meis2*^*f*lox/flox^; *Pax2*^Cre/+^ animals show a similar mutant cochlear phenotype to *Chd7*^flox/flox^; *Pax2*^Cre/+^ animals. The asterisk indicates the ending of the cochlear duct. **(D)** Distribution of Meis2 binding sites by their position with respect to the nearest transcription start site (TSS) in VOT-E36 cells. **(E)** Density plots showing the distribution of Meis2 binding sites vs. input control in VOT-E36 cells. **(F)** Input control and Meis2 ChIP-seq read profiles from VOT-E36 cells. Detected Meis2 binding sites within or close to the genomic regions of *Pax2, Pax8, Six1* and *Six4* are boxed.

In order to identify potential target genes of Meis2 in the mammalian cochlea, we performed a microarray-based screen for differential gene expression in *Meis2*^flox/flox^; *Pax2*^Cre/+^ mutant vs. wild-type cochleas (for details, see section “Materials and Methods”). We used whole E15 cochleas when *Meis2* expression is detected in the cochlear duct, the spiral ganglion and the auditory nerve (Figure 1E). The results of the microarrays showed that the vast majority of the 255 transcripts differentially expressed were downregulated in mutant cochleas (Figure 6A). Several of the downregulated genes are expressed in the cochlear duct or the spiral ganglion (Table 1). Within this group of genes, the cochlea of mouse mutants for the chromatin remodeling enzyme *Chd7* has been described to undergo an abnormal twist at their apex (Hurd et al., 2010). We first confirmed downregulation of *Chd7* by qPCR (Figure 6B) and then created a conditional mouse mutants for *Chd7* using the *Pax2*-Cre driver. Cleared whole mounts of inner ears isolated from *Chd7*^flox/flox^; *Pax2*^Cre/+^ mutants revealed a defective phenotype in cochlear coiling which was very similar to the one observed in *Meis2*^flox/flox^; *Pax2*^Cre/+^ mutants (Figure 6C). Therefore, *Chd7* may be a crucial downstream target of *Meis2*.

**TABLE 1:** Summary of genes downregulated in the cochlea of *Meis2* mutants.

Gene (MGI reference)	Fold Downregulation	Expression pattern
Acidic nuclear phosphoprotein 32 family (5332981)	4.1x	Mesenchyme
THUMP domain containing 3 (1277973)	3.7x	Otic capsule
Itchy, E3 ubiquitin protein ligase (4825634)	3.4x	Ganglion
Zinc finger protein 788 (1914857)	3.1x	Ganglion
Neurofilament, light polypeptide (97313)	3.1x	Ganglion
Trafficking protein particle complex 4 (4480575)	2.9x	Ganglion
Centrosome/spindle pole associated protein (2681832)	2.7x	Ganglion
FK506 binding protein 3 (5335642)	2.7x	Mesenchyme
Chromodomain helicase DNA binding protein 7 (2444748)	2.6x	Cochlear duct, ganglion and mesenchyme
Craniofacial development protein 1 (1344403)	2.4x	Cochlear duct and ganglion
Formin binding protein 1-like (1925642)	2.4x	Ganglion
Ephrin A1 (103236)	2.4x	Cochlear duct
Thymidylate synthase (98878)	2x	Mesenchyme

**The localization of the genes within the cochlea is annotated according to Mouse Genome Informatics (MGI) using RNA in situ hybridization. The table shows a list of genes which are expressed in the cochlea and down regulated by twofold or higher in Meis2 mutants according to the results obtained by the microarrays.**

To identify direct targets of Meis2, we performed ChIPseq analysis (see section “Materials and Methods”) using the VOT-E36 cell line derived from the ventral portion of the otic vesicle, the domain that gives rise to the cochlea (Lawoko-Kerali et al., 2004; Figure 6E). We identified a total of 2,401 Meis2 binding sites in the genome and a collection of 917 genes with a transcription start site closest to any Meis2 binding site (GEO GSE166072). As previously reported for Meis1 (Penkov et al., 2013; Marcos et al., 2015), most Meis2 binding sites were located in regions remote from their closest associated transcription start site (Figure 6D). Meis proteins select two main sequences in DNA: a motif resembling the Pbx-Hox binding sequence (A/TGATNNAT), to which it binds indirectly, and a direct binding site (TGACAG) (Penkov et al., 2013). Transcription factor motif enrichment analysis using Homer and MEME suite software (see section “Materials and Methods”) confirmed these motifs as the most frequently detected with 15.7 and 28.9% of peaks containing at least one of the conserved and/or the PBX-Hox binding sites, respectively. Gene ontology analysis revealed the presence of Meis2 binding peaks in the vicinity of 30 genes related to inner ear development, such as members of the *Pax*- and *Six*-gene families (Figure 6F). Interestingly, a Meis2 peak was observed in the vicinity of the *Otx1* gene, whose inactivation also leads to defective cochlear coiling (Morsli et al., 1999). A comparison between the observed peaks and differentially expressed genes in the cochlea of *Meis2*^flox/flox^; *Pax2*^Cre/+^ mutants at E15 revealed no Meis2 binding sites in the vicinity of the *Chd7* gene. However, an overlap in the vicinity of 7 genes including *Dlg1, Pnn, Nsmce2, Usf3, Zfp945, Lrrcc1, Rybp* and *Mras* was observed. Interestingly, *Dlg1* is expressed in the otic vesicle and its loss is associated with a shortening of cochlear length (Iizuka-Kogo et al., 2015), albeit to a lesser degree than that observed in *Meis2*^flox/flox^; *Pax2*^Cre/+^ mutants. Therefore, loss of *Dlg1* expression may, at least in part, contribute to the shortening of the cochlear duct in *Meis2*^flox/flox^; *Pax2*^Cre/+^ mutants.

## Discussion

*Meis2* was expressed in all three tissue layers involved in otic induction: the endoderm, mesoderm and the neural tissue of the hindbrain. Correlating with the highest expression levels, the loss of *Meis2* in the hindbrain led to a phenotype similar to that observed by global inactivation at E9 (compare Figures 2A–D with Figures 3C–H). Therefore, it may be concluded that *Meis2* expression in the hindbrain is likely to be sufficient to account for *Meis2* contribution to otic vesicle formation. Genes required for inner ear formation, which are directly or indirectly induced by *Meis2* showed no changes in their expression in the hindbrain, and thus so far indicate no major defects in patterning within the hindbrain in *Meis2* mutants. However, yet unknown downstream targets for Meis2 within the hindbrain may be involved in inner ear induction. During later stages, otic vesicle development was also affected in *Meis2*^flox/flox^; *Foxg1*^Cre/+^ mutants that lacked *Meis2* expression in the otic placode and the neighboring mesoderm and endoderm. The otic vesicle was reduced at E11 and at the postnatal stage the inner ear showed severe defects (Figures 3I–L). Compared to *Meis2*^flox/flox^; *Pax2*^Cre/+^ mutants which show Cre activity only in the otic placode (Supplementary Figure 1 and Figure 5), *Meis2*^flox/flox^; *Foxg1*^Cre/+^ mutants show a more severe inner ear phenotype (Figures 3K,L). Therefore, inactivation of *Meis2* expression from the mesoderm and/or endoderm leads to additional defects during inner ear development. Within the inner ear, *Meis2* initially showed expression in the dorsal portion of the otic vesicle and was later on found in non-sensory tissue of the cochlea and the peripheral nervous system innervating the inner ear. Very similar patterns of expression for *Meis2* have also been described during chicken inner ear development (Sanchez-Guardado et al., 2011).

*Meis2*^flox/flox^; *Pax2*^Cre/+^ mouse mutants lacking *Meis2* throughout inner ear development showed a smaller otic vesicle at E10.5, indicating that initial expression of *Meis2* in the otic placode or vesicle is also required for its normal formation. Nevertheless, otic markers related to neurosensory development and formation of the cochlear anlage were unaffected. However, by E13.5 *Meis2*^flox/flox^; *Pax2*^Cre/+^ mutants a striking defective outgrowth of the cochlear duct became apparent which resulted in its abnormal coiling. The phenotype was similar to that described in mouse mutants lacking β-catenin, required for Wnt signaling, or the transcription factors *Sox9* or *Tbx1* (Xu et al., 2007; Trowe et al., 2010; Bohnenpoll et al., 2014). However, in mutants lacking *Sox9* or *Tbx1*, the observed inner ear phenotype is caused by the inactivation of these genes in the surrounding mesenchyme, pointing to their indirect influence on morphogenesis. Defective turning of the cochlear duct has also been described in a mouse mutant lacking the chromatin remodeling enzyme *Chd7* throughout inner ear development (Hurd et al., 2010). Interestingly, we found that *Chd7* expression was reduced in the cochlea of *Meis2*^flox/flox^; *Pax2*^Cre/+^ mutants, suggesting that these genes may share a common pathway. Indeed, inactivation of *Chd7* using the *Pax2*-Cre line led to a phenotype very similar to *Meis2*^flox/flox^; *Pax2*^Cre/+^ mutants. Moreover, like *Meis2*, *Chd7* null mutants also show severely reduced sized otic vesicles (Hurd et al., 2007). In spite of this functional relationship, we did not find evidence for the binding of Meis2 within the regulatory regions of *Chd7* in the VOT-E36 cell line. Additionally, *Chd7* mutants show reduced expression of *Ngn1*, which was unaltered in *Meis2* mutants in the present study. This suggests that *Chd7* and *Meis2* rather belong to parallel pathways controlling cochlear coiling.

Only very few genes (Hayashi et al., 2002) from the ChIPseq analysis showed binding close to the transcription start site of genes. Similar findings have been recently reported for Meis1 (Penkov et al., 2013; Marcos et al., 2015). The most frequently detected binding motif detected in our studies corresponded to a site to which Meis binds indirectly to DNA. A major group of binding partners that facilitate indirect contact with DNA belong to the Hox gene family. Due to its rostral position in the embryo, the inner ear is a relatively Hox-free tissue, although the expression of PG-1 group genes like *Hoxa1* and its control of downstream targets have been reported in the inner ear (Makki and Capecchi, 2010, 2011). Additionally, Meis cooperates with a wide variety of transcription factors containing homeodomains, such as members of the *Pax, Dlx*, and *Otx* gene families which are also expressed in the inner ear (Schulte and Geerts, 2019). Nevertheless, only very few genes with Meis2 binding sites showed an overlap with differentially expressed transcripts in the cochlea. Therefore, the VOT-E36 cell line used for the ChIPseq analysis might not reflect an ideal model for *in vivo* inner ear development. Unfortunately, ChIP analysis from isolated otic vesicles was not feasible in our hands owing to technical limitations due to small-scale tissue samples. Nevertheless, further analysis of the genes showing both differential expression in *Meis2*^flox/flox^; *Pax2*^Cre/+^ mutants and containing binding sites detected by the ChIPseq analysis may reveal novel regulators of cochlear morphogenesis which are controlled by Meis2.

## Materials and Methods

### Transgenic Mice

Mice carrying a floxed *Meis2* allele (Delgado et al., 2020), a floxed *Chd7* allele (Hurd et al., 2007), the *Sox2*-Cre (Hayashi et al., 2002), *Pax2*-Cre (Ohyama and Groves, 2004), *Foxg1*-Cre (Hebert and McConnell, 2000) and *MafB*-Cre (Wu et al., 2016) transgenes and the *ROSA26* Cre reporter strain (Soriano, 1999) have been described previously. With the exception of the *Meis2*^flox/flox^; *MafB*^Cre/+^ mutants which showed a penetrance of 50%, all other phenotypes were fully penetrant and observed in a minimum of *n* = 3 animals for each experimental condition. Experiments conformed to the institutional and national regulatory standards concerning animal welfare.

### β-Galactosidase Staining and *in situ* Hybridization

β-Galactosidase staining, RNA whole-mount *in situ* hybridization and sectioning of stained embryos have been described previously (Alvarez et al., 2003). Riboprobes were generated for detection of *Meis2* (Delgado et al., 2020), *LFng, Ngn1, Dlx5*, (Vazquez-Echeverria et al., 2008), *Fgf3, Pax2, MafB* (Alvarez et al., 2003) and *HoxB1* (Vendrell et al., 2013).

### Immunohistochemistry

For immunohistochemistry cryostat sections were prepared and processed using standard protocols. The following antibodies were used: Meis-2 (Mercader et al., 2005); Pax2 (PRB-276P) from Covance, Sox2 (sc-17320) from Santa Cruz Biotechnology; myosin VIIA (25-6790) from Proteus; calretinin (7699/3H) from Swant; cyclin D1 (RM-9104-S0) from Thermo Fisher Scientific. For immunofluorescence, cryostat sections were incubated with primary antibodies, and the corresponding secondary antibodies used were donkey anti-goat Alexa Fluor-488, and goat anti-rabbit Alexa Fluor-568 (all from Invitrogen). Some of the sections were counterstained with DAPI. Whole-mount immunolabeling, dehydration, and clearing of inner ears was performed as described previously (MacDonald and Rubel, 2008). Bright-field images were captured with a DFC 490 camera (Leica) on a Labophot-2 microscope (Nikon). Immunofluorescence images were taken with a Nikon Eclipse 90i fluorescence microscope, or Leica SP confocal microscope.

### Screening for Differentially Regulated Genes in *Meis2*^flox/flox^; *Pax2*^Cre/+^ Mutants

RNA was isolated from E15 cochleas of wild type and *Meis2*^flox/flox^; *Pax2*^Cre/+^ mutants using the RNeasy Mini Kit (Qiagen) according to the manufacturer’s instructions. RNA integrity was assessed using Agilent 2100 Bioanalyzer (Agilent). Labeling and hybridizations were performed according to protocols from Affymetrix. Briefly, 100–300 ng of total RNA were amplified and labeled using the WT Expression Kit (Ambion) and then hybridized to Mouse Gene 1.0 ST Arrays (Affymetrix) covering a total of 21,041 gene transcripts. Washing and scanning were performed using the Affymetrix GeneChip System (GeneChip Hybridization Oven 640, GeneChip Fluidics Station 450 and GeneChip Scanner 7G). The robust microarray analysis algorithm was used for background correction, intra- and inter-microarray normalization, and expression signal calculation. The absolute expression signal for each gene was calculated in each microarray and significance analysis of microarrays was applied to calculate differential expression and find the gene probe sets that characterized the highly metastatic samples. The method uses permutations to provide robust statistical inference of the most significant genes and provides > *P*-values adjusted to multiple testing using false discovery rate. Probe synthesis, hybridizations and microarray data analysis were performed by the Genomics facility of the Centro de Investigación del Cancer (Salamanca, Spain). The microarray data from this screen have been deposited at GEO with accession number GSE149916. Genes twofold or greater were examined for their expression in the cochlea (Visel et al., 2004; Diez-Roux et al., 2011; Agoston et al., 2014; Bult et al., 2019) and are listed in Table 1.

### Quantitative PCR

Individual RNA samples were each prepared from the cochleae of two embryonic day (E) 15 mice (i.e., 4 cochleae were used to generate one RNA sample). RNA was extracted following homogenization in TRIzol^®^ Reagent (Invitrogen), and according to manufacturer’s instructions. Each RNA was then reverse transcribed into cDNA using a High Capacity cDNA Reverse Transcription Kit (Applied Biosystems, Life Technologies). cDNA samples were amplified in a LightCycler^®^ 480 II (Roche Molecular Diagnostics, Pleasanton, CA, United States) using SYBR^®^ Green PCR Master Mix (Life Technologies); duplicate reactions were carried out with each cDNA sample. The thermocycling conditions consisted of an initial denaturation step of 10 min at 95°C, followed by 40 cycles at 95°C for 15 s and 60°C for 1 min. The sequences of the PCR primers used in this work were: *Gapdh*, TCCTGCACCACCAACTGCTT and GTGGCAGTGATGGCATGGAC; *Chd7*, GAATACCCCA CAGAAAGTGCC and TCGCTCTTCACTAGCTGAGCG. Data were analyzed using the Software version LCS480 1.5.0.39. Relative levels of mRNA expression were calculated according to the 2^–ΔΔCt^ method, using *Gapdh* as the housekeeping gene. The data presented are the results from three independent experiments.

### ChIP-Seq

US/VOT-E36 cells (University of Sheffield/ventral otocyst-epithelial cell line clone 36) were used, derived from the otocyst of Immortomice at embryonic day 10.5 (Lawoko-Kerali et al., 2004). The cells were cultured in minimum essential medium (Gibco) supplemented with 10% fetal bovine serum (Gibco) and 50 U/ml γ-IFN (Immunotools) in a humidified atmosphere with 10% CO_2_ and at a temperature of 33°C, conditions under which these cells proliferate in the absence of differentiation.

For the ChIP assay, once the cultures had reached 85–90% confluence, the chromatin from the cells in six 75-cm^2^ flasks was cross-linked in 1% formaldehyde for 10 min at room temperature; next, the reaction was quenched by adding glycine to a final concentration of 125 mM and mixing during an additional 5 min. Following removal of the medium and various washes with phosphate buffer saline, cells were scraped off the tissue culture flasks, pelleted and lysed; the chromatin was then sheared into 200–500 bp fragments by sonication (using pulses of 30 s on and 30 s off in a water-bath sonicator). Any remaining cellular debris was subsequently removed by centrifugation and the sonicated chromatin was pre-cleared for 4 h at 4°C with protein A agarose beads (Roche). After this time, the beads were removed by centrifugation; half the volume of the chromatin sample was immunoprecipitated (IP sample) at 4°C overnight with a 1:1 mixture of an antibody against Meis2 (K846) and K830, an antibody that recognizes both Meis 1a and Meis 2a isoforms (Penkov et al., 2013). A volume that was equivalent to one tenth of the volume used for the IP reaction was also at that time set apart and frozen, constituting the Input control sample. Following immunoprecipitation with the antibodies, beads that had been pre-blocked at 4°C overnight with BSA and a rabbit IgG isotype control (ChromPure Rabbit IgG, Jackson ImmunoResearch Laboratories) were added to each tube; the samples were then incubated at 4°C for 4 h. Afterward, a series of washes using multiple buffers were conducted in order to remove any molecule that had bound non-specifically to the beads; the chromatin was finally eluted from the beads and cross-linking to the antibody reversed by incubating the IP sample in the presence of NaCl at 65°C overnight; the Input sample that had been stored at –20°C was also incubated in the same solution at 65°C. The next day, both the IP and the Input chromatin were purified using a PCR purification kit (Qiagen), following manufacturer’s instructions. The samples were then used to carry out ChIP-seq analysis.

0.5 ng of total DNA for both Input and IP were used to generate barcoded libraries using the *NEBNext*^®^ *Ultra^TM^ II DNA Library Prep Kit for Illumina* (New England Biolabs). Basically, adapters were ligated to DNA followed by an amplification and clean up. The size of the libraries was checked using the Agilent 2100 Bioanalyzer High Sensitivity DNA chip and their concentration was determined using the Qubit^®^ fluorometer (Thermo Fisher Scientific).

Libraries were sequenced on a HiSeq 2500 (Illumina) and processed with RTA v1.18.66.3. FastQ files for each sample were obtained using bcl2fastq v2.20.0.422 software (Illumina).

Sequencing reads were trimmed for Illumina adapter sequence with cutadapt, aligned to the mouse reference genome (mm10 v92) with bowtie and PCR duplicates were excluded with samtools MarkDuplicates tool.

Peaks were called with HOMER2 and params “-region -localSize 50000 -size 150 -minDist 1000-ntagThreshold 5-regionRes 6.” Peaks were also annotated with HOMER2 and inspected for MOTIFs with Meme (from Meme Suite) and with HOMER2. Data have been deposited in the NCBI GEO database under accession number GSE166072.

## Data Availability Statement

The datasets presented in this study can be found in online repositories. The names of the repository/repositories and accession number(s) can be found below: https://www.ncbi.nlm.nih.gov/, GSE166072, https://www.ncbi.nlm.nih.gov/, GSE149916.

## Ethics Statement

The animal study was reviewed and approved by Ethics Committee of the University of Valladolid.

## Author Contributions

TS and MD designed the research. TS, MD, VV, IL-H, FG, EV, LC, and GG performed the research. MA, MT, and DM contributed unpublished reagents and analytic tools. TS, MD, VV, and EV analyzed the data. TS wrote the first draft of the manuscript and wrote the manuscript. TS, MD, DM, and FG edited the manuscript. All authors contributed to the article and approved the submitted version.

## Conflict of Interest

The authors declare that the research was conducted in the absence of any commercial or financial relationships that could be construed as a potential conflict of interest.

## Supplementary Material

Supplementary Figure 1 Expression domains of Cre lines. *MafB*-Cre is expressed in the neural tube (nt) corresponding to rhombomeres 5 and 6 of the hindbrain whereas *Pax2*-Cre is active in the otic placode (op). Next to the otic placode, *Foxg1*-Cre is also active in the peri-otic mesoderm (m) and endoderm (e).
