# Supplementary figures and images for: Meis2 Is Required for Inner Ear Formation and Proper Morphogenesis of the Cochlea
Source: Front Cell Dev Biol. 2021 May 28;9:679325. doi: 10.3389/fcell.2021.679325 (PMC8194062; doi:10.3389/fcell.2021.679325)

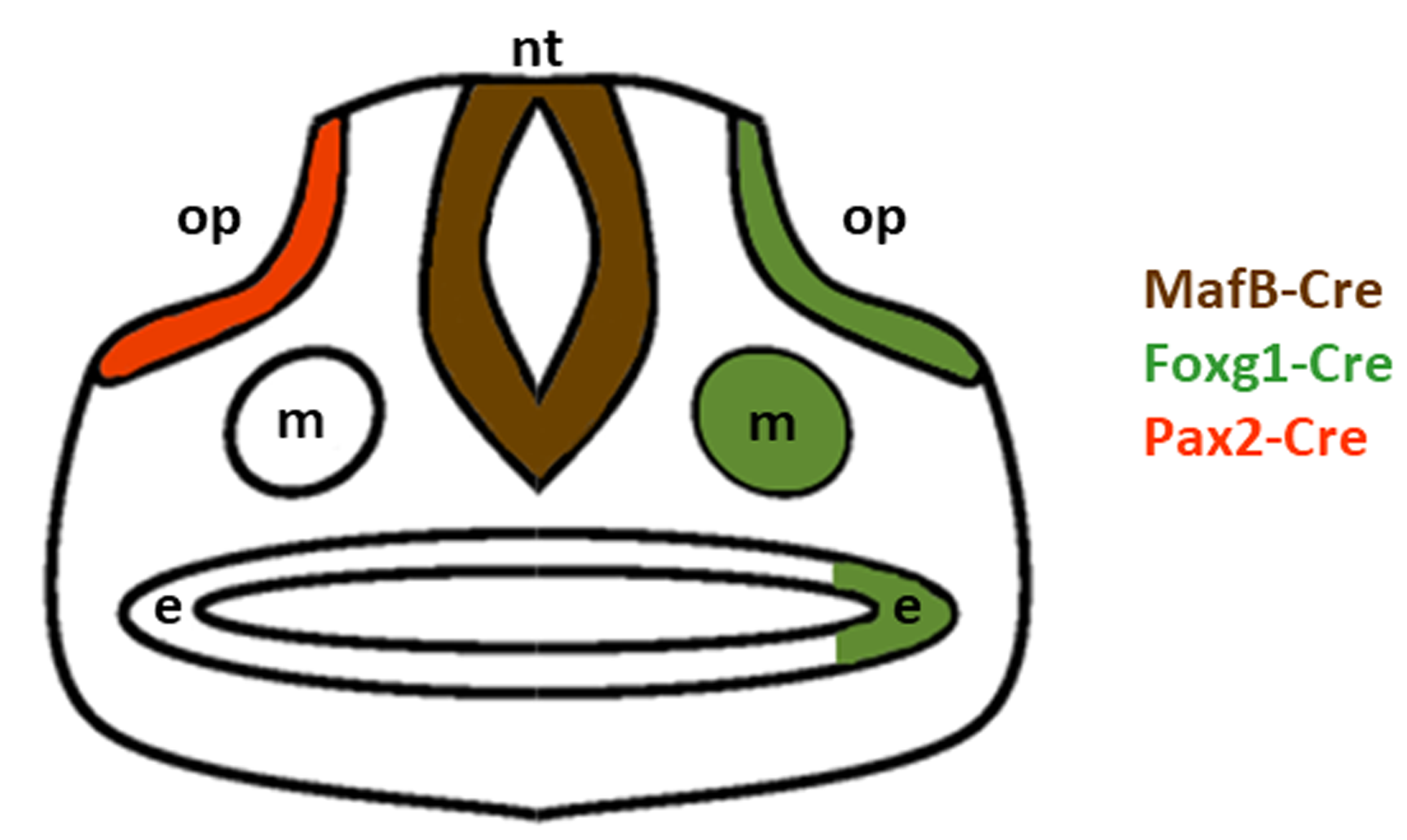

Supplement: Supplementary Figure 1 — Expression domains of Cre lines. MafB-Cre is expressed in the neural tube (nt) corresponding to rhombomeres 5 and 6 of the hindbrain whereas Pax2-Cre is active in the otic placode (op). Next to the otic placode, Foxg1-Cre is also active in the peri-otic mesoderm (m) and endoderm (e). [file Image_1.tif]
